# One-Year Clinical, Microbiological and Immunological Results of Local Doxycycline or Antimicrobial Photodynamic Therapy for Recurrent/Persisting Periodontal Pockets: A Randomized Clinical Trial

**DOI:** 10.3390/antibiotics11060738

**Published:** 2022-05-30

**Authors:** Raluca Cosgarea, Christoph A. Ramseier, Søren Jepsen, Nicole Birgit Arweiler, Pia Merete Jervøe-Storm, Ionela Batori-Andronescu, Ralf Rößler, Torsten Conrad, Sigrun Eick, Anton Sculean

**Affiliations:** 1 Department for Periodontology, Operative and Preventive Dentistry, University of Bonn, 53111 Bonn, Germany; soeren.jepsen@ukbonn.de (S.J.); storm@uni-bonn.de (P.M.J.-S.); 2 Clinic for Periodontology and Peri-Implant Diseases, Philipps University Marburg, 35033 Marburg, Germany; arweiler@med.uni-marburg.de; 3 Department of Prosthodontics, Iuliu Hatieganu University Cluj-Napoca, 400006 Cluj-Napoca, Romania; 4 Department of Periodontology, School of Dentistry, University of Bern, 3010 Bern, Switzerland; christoph.ramseier@zmk.unibe.ch (C.A.R.); sigrun.eick@zmk.unibe.ch (S.E.); anton.sculean@zmk.unibe.ch (A.S.); 5 Department, Periodontal Private Practice Cosmedica, 400185 Cluj-Napoca, Romania; andronescu.ionela@gmail.com; 6 University for Digital Technologies in Medicine and Dentistry, 9516 Wiltz, Luxembourg; ralf.roessler@dtmd.eu (R.R.); torsten.conrad@dr-conrad.de (T.C.); 7 Clinic for Mouth, Jaw and Plastic Facesurgery, University of Frankfurt, 6059 Frankfurt, Germany; 8 Private Practice, 55411 Bingen am Rhein, Germany

**Keywords:** supportive periodontal therapy, photodynamic therapy, local drug delivery, periodontal treatment, persistent periodontal pockets

## Abstract

We evaluated, in this study, the clinical, microbiological and immunological effects of local drug delivery (LDD) or photodynamic therapy (PDT), adjunctive to subgingival instrumentation (SI) in persistent or recurrent periodontal pockets in patients enrolled in supportive periodontal therapy (SPT) after one year. A total of 105 patients enrolled in SPT with persistent/recurrent pockets were randomly treated with SI +PDT or SI + LDD or SI (control). The number of treated sites with bleeding on probing (n BOP+), probing pocket depths (PPD), clinical attachment level (CAL), full-mouth plaque and bleeding scores (gingival bleeding index, %bleeding on probing-BOP) was evaluated at baseline and after 12 months. Additionally, eight periodontopathogens and the immunomarkers IL-1β (interleukin)and MMP-8 (matrix metalloprotease) were quantitatively determined using real-time PCR and ELISA, respectively. All three treatments resulted in statistically significant clinical improvements (*p* < 0.05) without statistically significant intergroup differences (*p* > 0.05), which were maintained up to 12 months. The presence of BOP negatively affected the PPD and CAL. Moreover, statistically significantly fewer bleeding sites at 12 months were observed in the test groups (*p* = 0.049). Several periodontopathogens were reduced after 12 months. In conclusion, the present data indicate that in periodontal patients enrolled in SPT, treatment of persistent/recurrent pockets with SI alone or combined with either PDT or LDD may lead to comparable clinical, microbiological and immunological improvements, which are maintained up to 12 months. Secondly, the presence of BOP directly impacts the PPD and CAL.

## 1. Introduction

Periodontal patients require a lifetime of periodontal maintenance therapy, also known as step 4 periodontal therapy or supportive periodontal therapy (SPT), after finalizing active periodontal treatment (Step 1–3 periodontal therapy according to the EFP S3 Clinical Therapy Guidelines) [1]. In this phase of the treatment, sites at risk for disease progression with probing depths (PD) of ≥4 mm and bleeding on probing (BOP) are diagnosed as persistent or recurrent periodontal pockets, and are re-treated in order to prevent the recurrence of disease and stop its progression [1,2,3,4]. In each SPT session, a re-assessment of the periodontal status and self-performed oral hygiene is conducted, followed by the re-instrumentation of persistent/recurrent periodontal pockets, re-enforcement of the self-performed oral hygiene and a supragingival professional tooth cleaning. Encompassing this regular professional mechanical biofilm removal in SPT sessions, it has been proven to be effective in assuring limited clinical attachment level changes and low rates of tooth loss (0.09–0.15 weighted mean yearly tooth loss rate for a period of 5–14 years) [5,6,7].

Despite the fact that mechanical removal of supra and subgingival soft and hard biofilm tooth deposits with mechanical/manual instruments still represents the gold standard for treatment of residual/recurrent periodontal pockets during SPT (EFP treatment guidelines Sanz et al. 2020), alternative/adjunctive treatments, such as the use of antimicrobials or antimicrobial photodynamic therapy (PDT), have been also evaluated [8,9,10,11,12,13].

PDT aims at inactivating the bacterial cells by exposure to light at a suitable wavelength. A photosensitizer, such as toluidine blue/methylene blue, which absorbs light, is previously bound to bacterial cells. Activating the light in the specific wavelength and in the presence of molecular oxygen produces cytotoxic singlet oxygen free radicals [14,15,16,17,18]. Various in vitro studies have proven activity in eliminating several periodontopathogens by combining toluidine blue with a helium/neon soft laser irradiation [16,19,20,21]. Additionally, promising clinical, microbiological, and immunological results were obtained when PDT was adjunctively used to subgingival instrumentation (SI) during Step 2 (“cause-related therapy”) [22,23] and Step 4 periodontal therapy (“supportice periodontal therapy”- SPT) [9,10,12]. On the other hand, other authors reported contradictory clinical and microbiological outcomes, both for active as well as for maintenance periodontal therapy [10,12,24,25,26,27,28]. Undisputable however, is the fact that PDT lacks the risk of developing bacterial resistance as well as genotoxic or mutagenic effects, as opposed to the systemic/local antimicrobials used on a long-term basis [29,30]. Thus, PDT therapy may be highly clinically relevant when treating periodontitis, a chronic biofilm-associated disease.

Similarly, the adjunctive application of local antimicrobials after SI has shown greater PD reduction and clinical attachment level (CAL) gains, as compared to mechanical debridement [31]. Of these, the local drug delivery (LDD) with 14% doxycycline in persistent/recurrent periodontal pockets seemed to exhibit comparable results to SI [32]. In maintenance therapy, locally applied doxycycline was also associated with clinical improvements on a short-term basis, even in teeth with furcation involvements [11].

Considering that antibiotic resistance is a worldwide increasing problem and that the repeated use of antibiotics represents its main cause, there is substantial concern about the repeated use of locally delivered doxycycline (LDD). Consecutively, alternative therapies such as PDT represent the scientific focus in periodontal treatment as compared to antibiotics.

However, only limited evidence is available for evaluating the clinical, microbiological and immunological effects of PDT and locally delivered antibiotics used adjunctively to SI, as compared to mechanical debridement alone, during maintenance periodontal therapy. In some studies, it seems that PDT or minocycline microspheres applied adjunctively to SI have resulted in comparable outcomes [33]. Corroborating data were published for peri-implantitis lesions, where similar outcomes after 12 months were observed for the reduction in mucosal inflammation for both PDT as well as the adjunctive application of minocycline microspheres [34].

Recently, we published 6 months of data from a randomized controlled clinical trial (RCT) for patients in SPT that were treated with PDT or LDD adjunctive to SI as compared to a control group receiving only mechanical debridement in persistent/recurrent periodontal pockets [35]. All three treatments showed statistically significant improvements. Several bacterial species were reduced in both PDT- and LDD-treated patients, however, with statistically significantly higher reductions after 6 months for LDD compared to PDT and the control group. 

The aim of this analysis was to evaluate, after 12 months, the clinical, microbiological and immunological effects of PDT or LDD applied adjunctively to SI as compared to SI alone in persistent/recurrent pockets of patients enrolled in SPT.

## 2. Results

**Figure 1:**
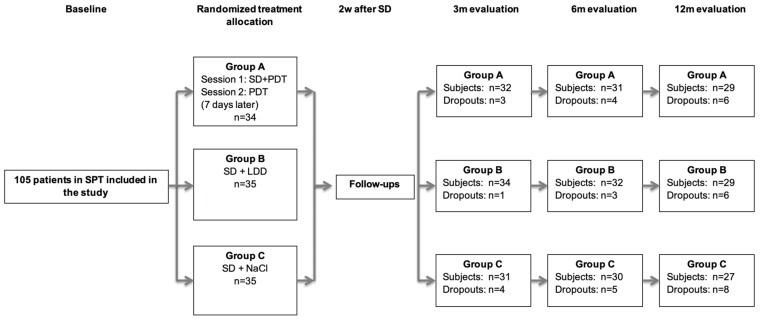
Flowchart of the study (FMPS: full-mouth plaque score; SI: subgingival instrumentation; LDD: local drug delivery; PDT: photodynamic therapy). SPT = supportive periodontal therapy; SD = subgingival debridement within 2 consecutive days; PDT = photodynamic therapy; LDD = locally delivered drug; m = months; d = days.

From the initially treated 105 patients (*n* = 35 per treatment group), 75 subjects (group A: *n* = 26; group B: *n* = 24; group C: *n* = 25) completed the evaluation at 12 months (Figure 1). Baseline demographics had been previously published (Table 1 in [35]). The age and gender distribution had been equally performed between the groups (*p* > 0.05). In all three treatment groups, the majority of the patients were diagnosed with stage III grade B periodontitis (Table 1 in [35]).

**Figure 2:**
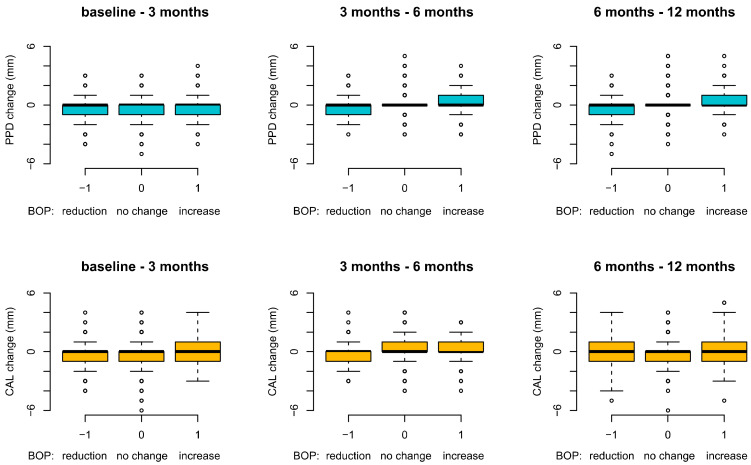
Site-based analysis: PPD and CAL changes in relation to BOP over 12 months (BOP: bleeding on probing; PPD: probing pocket depth; CAL: clinical attachment level).

The site-based analysis for all patients showed at 6 and 12 months a direct relationship between BOP and PPD change: the BOP increase was associated with a PPD increase, and vice versa, a BOP reduction led to a decrease in PPD (Figure 1). This can be observed by the fact that a PPD increase at 3–6 months and 6–12 months happens (the forest plot shifts in positive values on the vertical scale) when the BOP is present (“1” on the horizontal diagram line). Similar findings were observed for CAL changes at 3–6 months (Figure 2): the CAL plots are above “0”, indicating a CAL increase (CAL-loss) when BOP is “+”/”1”, and vice versa, when BOP is “−1”, the PPD and CAL plots are <”0”, indicating a PPD-reduction and a CAL-gain/stability.

**Figure 3:**
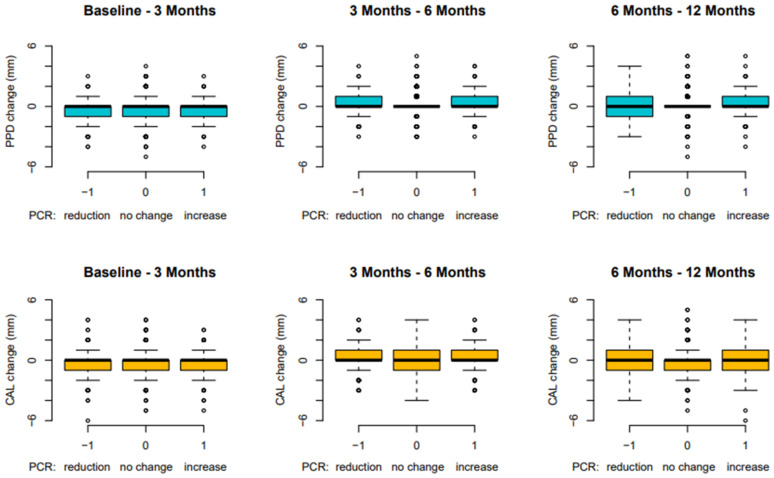
Site-based analysis: PPD and CAL changes in relation to FMPS over 12 months (FMPS: full-mouth plaque score; PPD: probing pocket depth; CAL: clinical attachment level).

On the other hand, plaque (FMPS) seemed to have no impact on PPD and CAL changes over 12 months (Figure 3).

Results of the site-based and full-mouth analyses at 12 months for the periodontal parameters PD, CAL, number of bleeding sites and plaque (FMPS) and gingival bleeding scores (GBI) can be found in Table 1. Except for GBI in the control group and FMPS in all groups that showed an increase at 12 months, all other evaluated parameters (PPD, CAL, n BOP+ sites, BOP) were reduced at 12 months (Table 1). No statistically significant differences could be observed between the groups at 12 months for PPD, CAL, BOP, FMPS and GBI (*p* > 0.05, Table 1). Statistically significant differences between the three treatment groups were shown for the number of bleeding sites at 12 months (*p* = 0.049).

Table 2 presents subgroup analyses for PPD and CAL values and changes over 12 months at the sites exhibiting no signs of plaque (PC -) or bleeding (BOP -) and those exhibiting both (PC + and BOP +). Patients without plaque or bleeding signs show improvements in PPD (PD reductions) and CAL (CAL-gain), whereas those with both BOP and PC show PPD-reductions and CAL gain only in the first 3 months. Thereafter, a slight mean PPD increase (up to 0.215 mm) and mean CAL-loss (up to 0.238 mm) can be detected (Table 2). At all timepoints up to 12 months, statistically significantly higher mean PPD and CAL values were noted for the sites with both BOP and PC (*p* < 0.0001).

Microbiological analyses of the eight determined periodontopathogens for up to 6 months were presented previously [35]. In the current analysis, we show the bacterial levels at 12 months. As pictured in Table 3, *Aggregatibacter actinomycetemcommitans, Treponema denticola* and *Filifactor allocis* remained reduced in all groups for up to 6 months. *Porphyromonas gingivalis* and *Tannerella forsythia* experienced a slight increase in the PDT group, whereas *C. rectus* increased in the LDD group. *Prevotella intermedia* and *Fusobacterium nucleatum* showed a slight increase at 12 months in all groups. Nonetheless, statistically significant differences favoring the LDD treatment were noted only for *T. denticola* (Table 3, *p* = 0.019).

IL-1β analysis shows a continuous slight decrease in the control group (group C), whereas in the other groups a slight increase can be seen at 12 months. MMP-8, however, showed a slight increase in all groups, with statistically significant differences favoring the control group only at 3 months (*p* = 0.024). Despite these various outcomes, no statistically significant differences between the three groups were found for any of the investigated inflammatory markers at 6 or 12 months (Table 4).

## 3. Discussion

In this study, we analyzed the 12 months clinical, microbiological and immunological effects of the adjunctive use of PDT or LDD in persistent/recurrent pockets of patients enrolled in SPT as compared to mechanical treatment alone.

One of the important findings in our study is that we have clearly shown the negative impact of BOP on the PPD and CAL: the sites presenting BOP show an increase in PPD and CAL loss at 6 and 12 months. Similarly, a BOP reduction resulted in a decrease in the PPD and CAL gain (Figure 2). The importance of BOP on the stability of pocket depths in maintenance therapy has been previously reported [4]. To the best of our knowledge, this is the first study that really shows this direct influence of BOP on the progression/stability of periodontal disease.

All clinical periodontal parameters of the treated sites (PPD, CAL, number of bleeding sites) showed improvements up to 12 months. Notably, the sites treated adjunctively with PDT or LDD had statistically significantly fewer bleeding sites at 12 months compared to those with mechanical treatment alone (*p* = 0.049). This is also an important clinically relevant outcome since bleeding in SPT is one of the major parameters indicating inflammation in periodontal pockets and consequently disease progression [4], and is in line with outcomes of other studies [10]. Additionally, comparing the 12-month data with those at 3 and 6 months, it seems that all clinical periodontal parameters remained stable without any obvious changes between the follow-up appointments (at 3 m: mean PPD for PDT: 2.72 ± 0.30 mm, for LDD 2.64 ± 0.26 mm, for mean n BOP+: PDT 1.21 ± 0.75, LDD 1.12 ± 0.68; at 6 m: mean PPD for PDT 2.76 ± 0.39 mm, for LDD 2.66 ± 0.28; for mean n BOP+: PDT 0.17 ± 0.12, LDD 0.16 ± 0.12) [35].

Our outcomes are comparable with data from a recent meta-analysis [38] (adjunctive treatment: CAL gain 0.79 ± 0.381 mm, PPD reduction 1.163 ± 0.40 mm) and with those from other studies with follow-up up to 12–24 months where the adjunctive treatment (PDT or LDD) had been repeated every 3 months [39,40]. Only a few studies evaluated the clinical outcomes after 12 months after adjunctive PDT in periodontal patients in maintenance therapy [39,41]. Despite the protocol differences, our results are in line with these studies. Lulic and coworkers applied PDT four times within 14 days and reported after 12 months comparable changes for PPD (test: −0.27 ± 0.43 mm; control: −0.07 ± 0.61 mm) and CAL (test: −0.009 ± 0.41 mm, −0.20 ± 0.61 mm) [41]. Contrary to our results (Table 1), higher percentages of BOP and PI at the experimental sites were reported at all time points (Lulic et al.: BOP from 95% to 77% at 12 m, PI from 77% to 59% at 12 m) [41]. This may be related to the fact that Lulic et al. included in the study patients with already high full-mouth plaque levels, whereas we included only patients with very good oral hygiene (baseline FMPS 18.43%, BOP 16.82%) [35]. Comparable CAL-, PPD-changes and FMPS values were also observed in Carvalho and coworkers (baseline-12 months changes for CAL test group: from 5.56 ± 0.83 to 4.60 ± 1.11 mm, PPD: 4.79 ± 0.47 mm to 3.55 ± 0.92 mm, FMPS: baseline 18.05 ± 23.95%, 12 m: 12.50 ± 17.68%) [39]. Interestingly, despite the fact that Carvalho et al. repeated the treatment at all follow-up appointments up to 12 months, BOP values were slightly higher (23.61 ± 21.82%) as compared to our study where the treatment has been performed only twice within 8 days at baseline. Both baseline and the 12 months mean values for PPD and CAL were higher in the abovementioned studies compared to ours [39,41]. This may be explained by protocol differences, such as the fact that in the analysis we included all sites at the treated tooth and not only those ≥4 mm, thus lower baseline mean values were calculated. Additionally, the subjects included in our study were stable maintenance patients with good oral hygiene and low levels of full-mouth bleeding scores and mostly diagnosed with stage III grade B periodontitis (*n* = 44) and we only had fewer subjects with advanced or rapidly progressing periodontitis forms (stage III grade C *n* = 19, stage IV grade B *n* = 10).

As stated in our previous publication [35], only a few options of locally delivered antibiotics exist and thus, limited evidence is provided on the efficacy of topically administered antibiotics in maintenance periodontal therapy [8,11,32,42,43,44].

Subjects receiving LDD showed comparable changes in CAL and PPD from baseline to 12 months with those in other studies administering LDD. Bogren et al. reported a relative AL gain at 12 months of 0.8 mm, and a PPD/full-mouth BOP change from 5.4 mm/52% at baseline to 4.5 mm/50% at 12 months [8]. Garett et al. obtained at 9 months, a CAL-gain of 0.72 ± 0.13 mm and a PPD-reduction of 1.28 ± 0.09 mm [45]. Despite the protocol differences in patient selection, treatment protocol (with or without mechanical debridement before application of LDD), or statistical analysis (some authors included in the analyses only a site ≥4 mm, whereas we considered mean values of all sites at the test teeth), our results corroborate these [5,8,39,40,41], also pointing out that no statistically significant additional benefits at the 12-month follow-up were observed between the LDD-treated subjects or those with mechanical debridement alone [8,11,44,45].

Altogether, compared to the results at 3 and 6 months, the microbiological results by trend showed a further decrease in the levels of all investigated microorganisms in all treatment groups at 12 months (Table 3). With the exception of *T. denticola*, which was significantly more reduced in the LDD group compared to the other treatment groups, no statistically significant differences were registered between the groups. As compared to baseline, at 12 months, *P. gingivalis*, *T. forsythia*, *P. intermedia*, *F. nucleatum* and *Campylobacter rectus* showed elevated levels to some degree in some treatment groups. This may be related to the increase in full-mouth plaque scores in all three groups, which is known to have a substantial impact on anaerobic subgingival flora.

Similar findings were observed by Rühling et al., Müller-Campanile et al. and Shaddox et al., who reported no statistically significant bacterial improvements [46,47,48].

The immunological analysis revealed a slight increase (*p* > 0.05) in the IL-1β and MMP-8 levels in all treatment groups at 12 months compared to baseline. This may be related to the increase in FMPS as well as the increase in marginal gingival bleeding (GBI-gingival bleeding index) in some groups. Nonetheless, considering overall that all patients maintained improvement in the clinical periodontal parameters (PPD, CAL, n sites with BOP+), as well as the reduced levels of periodontopathogens, this minor increase in immunomarkers may be insufficient to influence or indicate a progression of the disease.

As mentioned in our previous publication [35], one of the limitations of the present study represents the lack of stratification in the randomization procedures related to the clinical parameters or to smoking. However, except for baseline CAL, no statistically significant differences between the treatment groups were observed for the other parameters (Bonferroni corrections were applied).

Considering the increasing global problem of antimicrobial resistance, and with it, the need for reducing antibiotics, it seems relevant that future research may focus on alternative adjunctive treatments to non-surgical periodontal therapy, including paraprobiotics, probiotics and postbiotics, or various combinations of these with photodynamic therapy or ozone therapy [49].

## 4. Material and Methods

### 4.1. Study Subjects, Inclusion and Exclusion Criteria

This prospective, randomized, single-blinded clinical trial was planned and conducted according to the criteria set in the Declaration of Helsinki (1964, revision 2008), approved by the Ethical Committee of the Faculty of Medicine and Pharmacy of Cluj-Napoca (Application #390/02.07.2015) and registered in the ISRCTN trial registry (registration ID: ISRCTN17209965, https://www.isrctn.com/ISRCTN17209965; accessed on 26 February 2021). All study subjects were thoroughly informed about the protocol and were included in the study after signing the informed consent.

A detailed description of the clinical protocol was published previously [35]. Briefly, 105 periodontitis patients (periodontitis stages I–IV, grade A/B/C) enrolled in SPT were enrolled in this study. In order to be included, each had to present a minimum of four teeth with at least one site with a PD of ≥4 mm and bleeding (BOP+) or with a PD of ≥ 5 mm (defined as recurrent or persistent periodontal pockets). Additionally, subjects had to be in SPT for at least 6 months, over 35 years, with a good level of oral hygiene (plaque control record (FMPS) after O’Leary 1972 ≤30%) [37], and be systemically healthy. Exclusion criteria were pregnant subjects, local/systemic intake of antibiotics or other medication with possible interactions with doxycycline (e.g., coumarin derivates, containing alcohol derivates, 5-fluor-uracyl/disulfiram derivates, amprenavir oral solutions, lopinavir/ritonavir oral solution) or periodontium (Ciclosporin A, compounds of Phenytoin, calcium channel blockers) within the preceding six months. Patients smoking over 10 cigarettes/day were not included in the study. Medical and smoking history (smokers: patients smoking <10 cigarettes/day [50]), former smokers: stopped smoking 5 years before the beginning of the study) were assessed at the study recruitment visit and re-checked at each follow-up.

### 4.2. Clinical Protocol

All patients were clinically examined by one blinded and previously calibrated periodontist (I.B.A.). Calibration was performed by measuring twice (48 h apart) five patients’ periodontal pocket depths (PD) and clinical attachment levels (CAL). When these measurements were similar to the millimeter at >90%, calibration was accepted (Cohen’s Kappa analyses: mean intra-examiner reliability PD: 0.84, CAL: 0.81).

All patients were treated by a single experienced periodontist (R.C.) according to a computer-generated randomization list as follows (flowchart of the study is in Figure 1): PDT group A (SI plus PDT and 7 days later a further PDT session); LDD group B (SI and LDD) and the control group, Group C (only SI). Allocation concealment from before treatment was warranted by opaque envelopes. The patients, clinical examiner, therapist and statistician were blinded from the therapy allocation.

Clinical periodontal parameters (PD and CAL) were assessed before therapy (baseline), at 3, 6 and 12 months after therapy, at 6 sites per tooth (mm-scaled periodontal probe, PCPUNC 15; Hu Friedy^®^, Chicago, IL, USA) [35]. At the same appointments, bleeding on probing (BOP), gingival bleeding (GBI according to Ainamo and Bay 1975) [46], and a full-mouth plaque score (FMPS according to O’Leary 1972) [44] were also determined. Additionally, gingival crevicular fluid (GCF) and biofilm samples for immunological/microbiological analysis were obtained from the four deepest sites using sterile paper points.

SI was performed at all test teeth (PD ≥ 4 mm and bleeding (BOP+) or with PD ≥ 5 mm), with ultrasonic instruments (EMS, Piezon Master 700^®^, Switzerland). As previously described [35], patients were randomly treated as follows:

**Group A:** First PDT session: after the ceasing of bleeding (ca. 5 min after SI), PDT was applied at 6 sites of each test tooth (HELBO Blue Photosensitizer, Bredent medical, Senden, Germany, for 3 min, rinsing with sterile saline solution, exposure to laser light for 10 s -HELBO TheraLite Laser, HELBO 3D Pocket Probe, Bredent medical, Senden, Germany, with a wavelength of 660 nm, an output power of 100 mW).

The second session (after 1 week): PDT was repeated without SI at all test teeth.

**Group B**: LDD (Ligosan^®^, Heraeus Kulzer, Germany) was applied to the bottom of the pocket only at the test site (PD ≥ 4 mm and bleeding (BOP+) or with PD ≥ 5 mm) and patients refrained from interdental flossing/brushing for the following 10 days.

**Group C:** Test sites were rinsed with sterile saline solution.

### 4.3. GCF and Microbial Sampling and Analysis

The four deepest sites were isolated with cotton rolls and after supragingival biofilm was carefully removed with cotton pellets, a paper strip (Periopaper^®^, Oraflow manufacturer, New York, NY, USA) was held at the entrance of the periodontal pocket for 20 s; thereafter, a sterile paper point (ISO 40) was inserted for 20 s to the bottom of the periodontal pocket. GCF samples were pooled and stored at −70 °C while microbial samples were stored at −20 °C after pooling until assayed.

Using commercially available ELISA kits (R&D Systems Europe Ltd., Abingdon, UK), the host-derived biomarkers IL-1β and MMP-8 were determined as described in Cosgarea et al. [51]. The detection levels of the used test kits were pg IL-1β and 0.5 ng MMP-8/samples.

The periodontopathogens *A. actinomycetemcomitans*, *P. gingivalis*, *T. forsythia*, *T. denticola*, *Parvimonas micra*, *F. nucleatum*, *Camphilobacter rectus* (*C. rectus*) and *Filifactor allocis* (*F. allocis*) were determined using a real-time polymerase chain reaction (rtPCR) as previously described [51]. The detection level was at 100 bacteria/sample.

### 4.4. Statistical Analysis

As previously described [35], we included 30 subjects per treatment group in order to reach a study power of ≥85% for a *p* of 0.05 (statistically significant) and to detect a difference of 1 bleeding site (BOP positive) with a standard deviation of 1.3 [34,52].

We considered BOP the primary outcome variable, while PPD, CAL and the levels of *A. actinomycetemcomitans*, *P. gingivalis*, *T. forsythia*, *P. intermedia*, *T. denticola*, *F. nucleatum*, *C. rectus*, and *F. allocis* and IL-1β and MMP were secondary variables. An experienced periodontitis and statistician (CR) used RStudio (Version 1.3.1093, RStudio Team (2020). RStudio: Integrated Development Environment for R. RStudio, PBC, Boston, MA, USA, URL http://www.rstudio.com/, accessed on 8 March 2021) for the statistical analysis by means of Chi-square tests, Mann–Whitney tests, a Student’s *t*-test, Kruskal–Wallis tests, Friedman tests, and one-way analysis of variance (ANOVA). All *p*-values < 0.05 were statistically significant. Microbial and immunological analyses were performed using the statistical software program SPSS (IBM Corp. Released 2019. IBM SPSS Statistics for Windows, Version 26.0. IBM Corp, Armonk, NY, USA).

## 5. Conclusions

In conclusion, the present data indicate that in periodontal patients enrolled in SPT, treatment of persistent/recurrent pockets with SI alone or combined with either PDT or LDD may lead to comparable clinical, microbiological and immunological improvements which are maintained for up to 12 months. Secondly, the presence of BOP directly impacts PPD and CAL.

## Figures and Tables

**Table 1:** Mean values and standard deviations for site-based and full-mouth clinical parameters.

Variables	Group A	Group B	Group C	Group Comparisons
	(SI + PDT)	(SI + LDD)	(SI + NaCl)	*p*-Value
	Baseline: *n* = 35	Baseline: *n* = 35	Baseline: *n* = 35	
	12 m: *n* = 26	12 m: *n* = 24	12 m: *n* = 25	
**Site-based results**				
**PPD (mm)**				
Baseline	2.96 ± 0.30	2.94 ± 0.20	2.97 ± 0.24	0.764
12 m	2.77 ± 0.39 ^s^	2.70 ± 0.31 ^s^	2.72 ± 0.32 ^s^	0.437
**CAL (mm)**				
Base	3.67 ± 0.81	4.13 ± 0.97	3.66 ± 0.83	0.074
12 m	3.45 ± 0.89	3.89 ± 1.15 ^s^	3.51 ± 1.05 ^s^	0.957
**n BoP + sites**				
Base	1.71 ± 0.69	1.82 ± 0.78	1.92 ± 0.91	0.992
12 m	1.17 ± 0.72 ^s^	0.94 ± 0.54 ^s^	1.28 ± 0.99 ^s^	0.049 ^s^
**Full-mouth results**				
**BOP (%)**				
Base	16.52 ± 8.31	17.36 ± 9.33	16.57 ± 8.57	0.949
12 m	13.27 ± 8.41 ^s^	10.79 ± 5.59	13.45 ± 10.55 ^s^	0.704
**FMPS (%)**				
Base	18.32 ± 7.32	18.39 ± 6.20	18.57 ± 7.71	0.736
12 m	23.33 ± 12.68 ^s^	28.69 ± 15.77	23.23 ± 14.61	0.337
**GBI (%)**				
Base	2.56 ± 4.50	4.39 ± 6.75	4.07 ± 6.71	0.828
12 m	4.91 ± 8.56	3.47 ± 5.13	5.25 ± 5.44	0.290

Mean values and standard deviations for site-based and full-mouth parameters. Full-mouth parameters: intragroup comparisons with Friedman test; intergroup comparisons with Kruskal–Wallis test; PPD = probing pocket depth, CAL = clinical attachment level, BOP = bleeding on probing, GBI = gingival bleeding index [36], FMPS = full-mouth plaque score after O’Leary [37], m: months; base: baseline. (^s^) Statistically significant *p*-values (*p* < 0.05).

**Table 2:** Subgroup analysis: number of sites, mean PPD (mm) and CAL (mm) of sites with/without biofilm (PC) and bleeding (BOP) at baseline and after 3, 6 and 12 months.

	I: Absence of Both PC and BOP			II: Presence of Both PC and BOP			*p*-Value I versus II
	n	Mean	sd	n	Mean	sd	
**PPD**							
Baseline	1794	2.717	1.06	295	3.556	1.09	<0.0001 ^s^
3 m	1861	2.564	0.89	180	3.267	1.01	<0.0001 ^s^
6 m	1841	2.568	0.91	169	3.292	1.22	<0.0001 ^s^
12 m	1555	2.581	0.93	166	3.367	1.41	<0.0001 ^s^
Baseline—3 m	1861	−0.272	0.91	180	−0.178	0.88	0.4220
3–6 m	1841	−0.003	0.76	169	0.215	1.07	0.0050
6–12 m	1555	0.004	0.86	166	0.181	1.06	0.1000
**CAL**							
Baseline	1794	3.703	1.63	295	4.536	1.87	<0.0001 ^s^
3 m	1861	3.503	1.46	180	4.417	1.78	<0.0001 ^s^
6 m	1841	3.538	1.52	169	4.515	1.92	<0.0001 ^s^
12 m	1555	3.575	1.58	166	4.645	2.10	<0.0001 ^s^
Baseline—3 m	1861	−0.234	1.03	180	−0.156	1.06	0.5160
3–6 m	1841	−0.014	0.94	169	0.238	1.05	0.0070 ^s^
6–12 m	1555	−0.031	1.00	166	0.031	1.35	0.7860

The number of sites (*n*) with/without biofilm accumulation (PC) and signs of BOP, PPD and CAL (mean values and standard deviations) at follow-ups and their changes in-between. (^s^): Statistically significant *p*-value (*p* < 0.05).

**Table 3:** Mean counts (log10) ± SD of periodontal pathogens in all treatment groups (A: PDT, B: LDDs, C: control) at baseline and after 12 months (Kruskal–Wallis test, Friedman test).

Variables	Group A	Group B	Group C	Inter-Group *p*-Value
***A. actinomycetem-ecomitans* (log10)**				
Baseline	0.74 ± 1.67	0.68 ± 1.72	0.63 ± 1.79	0.899
12 m	0.15 ± 0.78	0.18 ± 0.90	0.14 ± 1.70	0.997
***P. gingivalis* (log10)**				
Baseline	3.45 ± 2.97	3.69 ± 2.97	3.84 ± 2.83	0.919
12 months	3.66 ± 2.95	2.26 ± 2.69	3.84 ± 2.76	0.084
***T. denticola* (log10)**				
Baseline	3.78 ± 2.96	3.06 ± 2.90	3.63 ± 2.50	0.611
12 m	**3.35 ± 2.88 ^s^**	**1.63 ± 2.28**	**2.38 ± 2.60**	**0.019 ^s^**
***T. forsythia* (log10)**				
Baseline	4.57 ± 2.91	4.72 ± 2.53	4.61 ± 2.68	1.000
12 m	5.30 ± 2.02	3.24 ± 2.95	4.56 ± 2.68	0.104
***P. intermedia* (log10)**				
Baseline	2.96 ± 3.00	2.19 ± 2.97	2.33 ± 2.88	0.481
12 m	3.18 ± 2.86	**3.51 ± 2.85**	3.13 ± 3.19	0.937
***F. nucleatum* (log10)**				
Baseline	6.87 ± 0.98	6.53 ± 1.91	6.49 ± 1.28	0.455
12 m	7.07 ± 1.14	6.81 ± 1.23	6.76 ± 1.17	0.354
***C. rectus* (log10)**				
Baseline	4.35 ± 2.78	4.15 ± 3.02	3.66 ± 3.02	0.534
12 m	3.68 ± 3.45	4.29 ± 2.90	3.53 ± 3.17	0.826
***F. allocis* (log10)**				
Baseline	5.23 ± 2.38	4.98 ± 2.60	4.92 ± 2.74	0.959
12 m	3.96 ± 3.37	3.84 ± 3.24	3.11 ± 3.35	0.392

(^s^): Statistically significant *p*-value (*p* < 0.05).

**Table 4:** Mean levels (±SD) of IL-1β and MMP-8 in the three treatment groups (A: PDT, B: LDDs, C: control) at baseline and after 3, 6 and 12 months (mean ± SD).

Variables	Group A	Group B	Group C	Group Comparisons
				***p* Value**
**IL-1β (pg/site)**				
Baseline	57.43 ± 73.06	56.05 ± 65.69	69.69 ± 87.35	0.875
3 m	77.91 ± 94.49	58.59 ± 57.99	67.74 ± 77.56	0.850
6 m	72.90 ± 86.81	33.71 ± 25.57	57.59 ± 43.51	0.126
12 m	**67.57 ± 99.35**	60.80 ± 78.19	56.86 ± 50.11	0.716
**MMP-8 (ng/site)**				
Baseline	10.53 ± 4.89	11.85 ± 4.16	11.11 ± 4.89	0.566
3 m	11.74 ± 4.59	11.57 ± 5.31	8.96 ± 4.43	0.024 ^s^
6 m	11.08 ± 4.55	12.82 ± 4.92	13.37 ± 6.38	0.294
12 m	11.45 ± 5.08	12.00 ± 3.89	11.49 ± 4.54	0.801

(^s^): Statistical significant *p*-values (*p* < 0.05). IL: Interleukin; MMP: matrix metallo protease.

## Data Availability

Data is available per request at raluca.cosgarea@gmail.com.
